# Severe mpox in an immunocompromised, non-traveller South African male

**DOI:** 10.4102/sajid.v41i1.814

**Published:** 2026-06-30

**Authors:** Preethi A. John, Omphemetse Matikane, Matilda Mphahlele, Barbara B. Makumbi

**Affiliations:** 1 Department of Internal Medicine, Tambo Memorial Hospital, Johannesburg, South Africa; 2 Department of Dermatology, Tambo Memorial Hospital, Johannesburg, South Africa

**Keywords:** mpox, immunocompromised host, atypical presentation, close contact transmission, household transmission, severe disease, HIV co-infection, non-traveller

## Abstract

Mpox is a zoonotic orthopoxvirus infection historically linked to being in endemic regions, travel and close physical contact, including within men who have sex with men (MSM) communities. We report a case of severe mpox in a heterosexual man with advanced human immunodeficiency virus (HIV) disease and no travel history to endemic areas. The patient developed extensive cutaneous disease and transmitted mpox to household contacts, illustrating non-sexual transmission. Management included supportive care and tecovirimat under Section 21 authorisation (a regulatory mechanism in South Africa permitting access to an unregistered medicine) with good clinical response.

**Contribution:**

This case highlights the changing epidemiology of mpox, the risk of severe disease in immunocompromised patients, household and non-sexual transmission. We also emphasise the importance of early diagnosis, infection control and antiviral therapy when indicated.

## Introduction

Mpox is a zoonotic disease caused by the Monkeypox virus (an orthopoxvirus), with clinical features similar to smallpox.^1,2,3^ Human mpox was first identified in the Democratic Republic of Congo in 1970. Since then, it has been mainly reported in Central and West Africa.^1,4^ Mpox has historically been associated with travel to endemic African regions and with men who have sex with men (MSM); however, transmission occurs primarily through close physical contact and is not inherently sexual.^1,2^ The case presented highlights a change in epidemiology. Our case looks at atypical transmission routes and severe disease presentation in an immunocompromised host outside a previously recognised high-risk group.

## Case presentation

**FIGURE 1:**
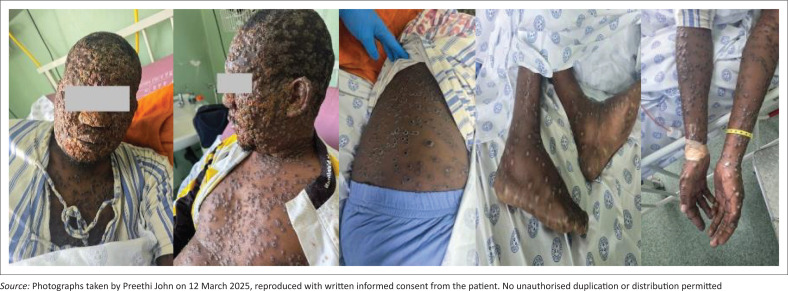
Lesions on presentation: Multiple papules, nodules and vesicles with central necrosis involving the whole body with facial swelling. Tense blisters were noted on the palms and soles. Herpetic erosion and secondary crusting were noted on the face.

A 38-year-old heterosexual male residing in Germiston, Johannesburg, presented to a nearby emergency centre on 08 March 2025 with a 3-week history of painful generalised rash, myalgia, headache, body weakness and rigours. He worked as a taxi driver transporting international travellers to and from OR Tambo International Airport. The patient was living with human immunodeficiency virus (HIV) (cluster of differentiation 4 [CD4] count 127 cells/µL, World Health Organization [WHO] Stage IV) on combination antiretroviral therapy (ART) (daily tenofovir 300 mg, lamivudine 300 mg and dolutegravir 50 mg in a fixed-dose combination). The rash began as two pruritic papules on the left antecubital fossa, subsequently spreading to the entire body, including his palms, soles, genital area and oral mucosa. He had no history of contact with rodents, primates or confirmed mpox cases. He denied MSM activity but had engaged in unprotected intercourse with his cisgender girlfriend 2 days before symptom onset. His partner subsequently developed similar lesions 1 week after his initial presentation. On examination, he appeared acutely ill with pyrexia (temperature of 40 °C), tachycardia (pulse of 124 beats per minute [bpm]), respiratory rate 20 breaths/min, blood pressure (BP) 124/88 mmHg and oxygen saturation 98% on room air. He had extensive lymphadenopathy involving the inguinal and cervical regions. Dermatological findings included multiple papules, nodules and vesicles with central necrosis involving the whole body with facial swelling. Similar tense blisters were noted on palms and soles. Herpetic erosion and secondary crusting were noted on the face as per Figure 1. Our differential diagnosis included: Mpox with secondary infection, secondary syphilis, a deep fungal infection, varicella zoster (chickenpox) and disseminated herpes simplex virus (HSV) infection.

## Investigations

Elevated inflammatory markers: CRP 218 mg/L, White Cell Count [WCC] 14.65 × 10^9^/L (as per Table 1).

**TABLE 1:** Biochemical profile.

Variable	Results on admission: 08 March 2025	Results during admission: 12 March 2025	Reference values
White cell count	14.65 × 10^9^/L	9.89 × 10^9^/L	3.92 – 10.40 × 10^9^/L
Haemoglobin	13.6 g/dL	11.6 g/dL	13.45 g/dL – 17.5 g/dL
Platelet count	474 × 10^9^/L	432 × 10^9^/L	171 – 388 × 10^9^/L
Sodium	127 mmol/L	135 mmol/L	136 mmol/L – 145 mmol/L
Potassium	4.7 mmol/L	4.2 mmol/L	3.5 mmol/L – 5.1 mmol/L
Urea	4.0 mmol/L	4.8 mmol/L	2.1 mmol/L – 7.1 mmol/L
Creatinine	80 µmol/L	55 µmol/L	64 µmol/L – 104 µmol/L
eGFR	107 mL/min/1.73 m^2^	126 mL/min/1.73 m^2^	> 60 mL/min/1.73 m^2^
Albumin	26 g/L	22 g/L	35 g/L – 52 g/L
Total Bilirubin	14 µmol/L	7 µmol/L	5 µmol/L – 21 µmol/L
Conjugated Bilirubin	8 µmol/L	4 µmol/L	0 µmol/L – 3 µmol/L
ALT	56 U/L	26 U/L	10 U/L – 40 U/L
AST	52 U/L	46 U/L	15 U/L – 40 U/L
GGT	137 U/L	48 U/L	< 68 U/L
ALP	248 U/L	109 U/L	53 U/L – 128 U/L
CRP	218 mg/L	169 mg/L	< 10 mg/L
PCT	-	0.40 µg/L	< 0.5 µg/L
Treponema pallidum antibodies	Reactive	-	-
RPR	Non-Reactive	-	-
Cryptococcal antigen	Negative	-	-
CD4	127 cells/µL	-	332 cells/µL – 1642 cells/µL
Hepatitis A IgM	-	Negative	-
Hepatitis B surface antigen	-	Negative	-
Hepatitis C antibody	-	Negative	-

*Source:* Compiled by the authors from patient laboratory records, 2025

CD4, cluster of differentiation 4; eGFR, estimated glomerular filtration rate; ALT, alanine aminotransferase; AST, aspartate aminotransferase; GGT, gamma-glutamyl transferase; ALP, alkaline phosphatase; CRP, C-reactive protein; PCT, procalcitonin; RPR, rapid plasma reagin.

**FIGURE 2:**
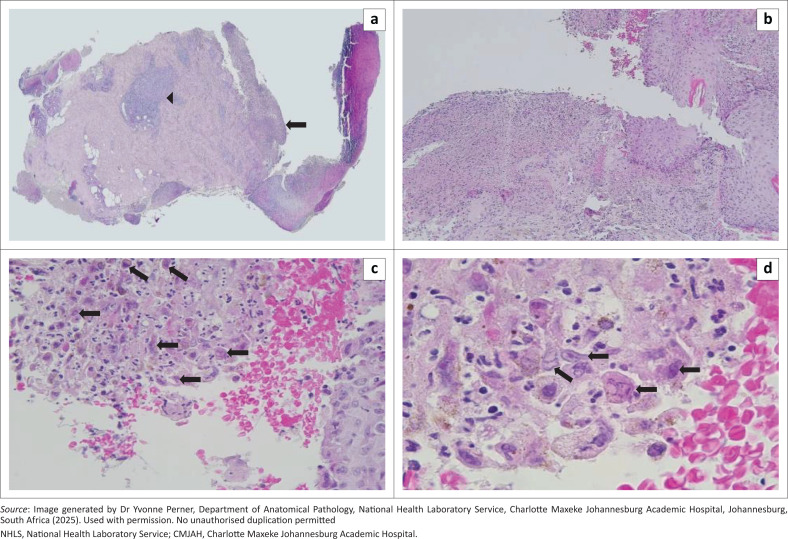
Histological evaluation of skin biopsy specimen. Image a: H&E (hematoxylin and eosin–stained histology slides) low power (20X) showing epidermal ulceration and intense inflammation of the ulcer base (arrow) and within the underlying dermis (arrowhead). Image b: H&E low power (100X) showing the ulcer base and intact, inflamed epidermis at the shoulder of the ulcer. Image c: H&E high power (400X) showing a viral cytopathy within epidermal squamous (arrow) adjacent to the ulcer. Image d: H&E high power (1000X) showing a viral cytopathy within epidermal squamous (arrow) adjacent to the ulcer. National Health Laboratory Service laboratory at Charlotte Maxeke Johannesburg Academic Hospital, 2025.

Mpox diagnosis confirmed by Polymerase Chain Reaction (PCR) from lesion swab. A skin punch biopsy on admission had features of a pustule, suggestive of a viral dermatosis. No fungal elements were identified on histological examination, and multinucleated giant cells or ballooning degeneration – features typically associated with varicella or herpes simplex virus infection were not present, making these diagnoses unlikely. The appearances were consistent with mpox as confirmed by PCR. Immunochemistry testing of both HSV and *Treponema pallidum* was negative (as per Figure 2).

**FIGURE 3:**
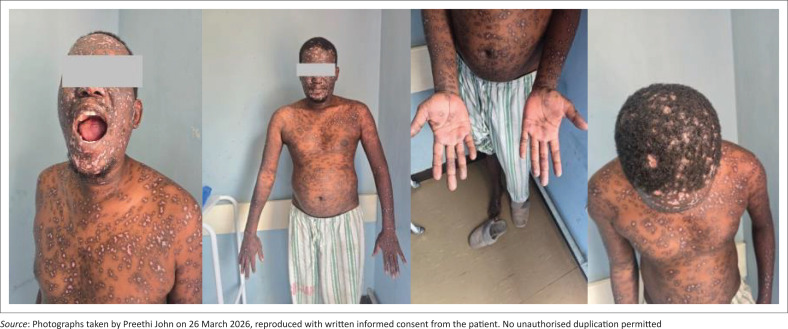
After 14 days of tecovirimat and 4 weeks of isolation at hospital: Hypopigmentation with scarring were noted with good clinical improvement.

The patient was initially empirically treated with intravenous ceftriaxone for suspected secondary bacterial infection of the extensively ulcerated and crusted skin lesions because of advanced HIV, fever and markedly elevated inflammatory markers (CRP 218 mg/L). Antibiotics were directed at bacterial superinfection of the cutaneous lesions rather than mpox, in keeping with the principle that antibacterials have no role in uncomplicated viral infection. Treatment was de-escalated to intravenous co-amoxiclav after consultation with an infectious diseases specialist. The patient continued ART, received intravenous fluids, oral feeds and wound care. As a result of his extensive rash and immunocompromised status, the patient required tecovirimat (antiviral for mpox). Tecovirimat was obtained following Section 21 authorisation (a regulatory mechanism by which the South African Health Products Regulatory Authority permits access to an unregistered medicine), and he was initiated on tecovirimat 600 mg bd for 14 days. The case was notified immediately, and contact tracing was commenced by the district health services. The patient reported no side effects from the treatment, and good clinical improvement was noted during and after treatment completion, as per Figure 3. The patient completed 14 days of tecovirimat and almost 4 weeks of isolation in the hospital. He was discharged on 01 April 2025 to complete the rest of the isolation period at home. Contact tracing identified mpox infection in both the patient’s girlfriend and his young niece. The girlfriend likely acquired the infection through sexual contact shortly before the patient’s symptom onset. The niece, who resided in the same household, was exposed through close non-sexual contact, including shared living spaces, towels and frequent physical interaction during the patient’s prodromal and rash phases. She developed lesions approximately 10–12 days after the patient’s symptom onset, consistent with the recognised incubation period. This pattern of household transmission to a paediatric, non-sexual contact emphasises that mpox transmission is not limited to sexual contact and can occur via shared fomites and close physical contact within a household.^5^

## Discussion

This case highlights key insights into the changing epidemiology and clinical presentation of mpox.

### Changing epidemiology and transmission patterns

Historically, mpox was linked with travel to endemic regions and MSM communities.^3^ However, an outbreak in 2022 highlighted that most of the patients did not travel to endemic African regions, suggesting possible previously under detected community transmission. A global analysis of the 2022–2023 outbreak reported that most cases occurred in males (96.4%), with a high proportion identifying as MSM (86.9%) and sexual contact as the predominant mode of transmission (68.7%).^6^ Children under 18 years still constitute nearly 40% of mpox cases in endemic African countries, supporting the importance of household and non-sexual transmission in these settings.^5^ Secondary household transmission accounted for up to 11% of cases in the 2023 outbreak, which emphasises the need for isolation protocols beyond sexual partners.^7^ Transmission may also occur through large respiratory droplets during prolonged close contact, vertical transmission and exposure to contaminated objects.^1,8,4^ Our patient was a heterosexual male with no travel history to endemic regions or involvement with MSM networks who subsequently transmitted mpox to family members, illustrating the broader and evolving transmission patterns beyond traditional risk groups.

### Severe disease in immunocompromised patients

Individuals with advanced HIV and CD4 counts < 200 cells/mm^3^ are more vulnerable to disseminated mpox with complications such as secondary bacterial infections, sepsis, prolonged viral shedding and hospitalisation.^9,10^ The 2022 multi-country mpox outbreak highlighted that most deaths occurred in untreated advanced HIV. Furthermore, it was apparent that patients with HIV and CD4 of 20 cells/mm^3^ – 350 cells/mm^3^ had more extensive rashes with secondary bacterial infections and longer disease course.^9^ Conversely, HIV positive patients receiving effective ART with higher CD4 counts and undetectable viral loads experienced fewer complications and improved outcomes.^1^ Our patient’s CD4 count of 127 cells/mm^3^ explains his extensive skin involvement, underscoring the importance of immune status in prognosis.

### Treatment considerations

The management of mpox is primarily supportive, but antivirals such as tecovirimat are recommended for patients with severe disease, atypical sites of infection or immunocompromised patients (e.g. advanced HIV [CD4 < 200 cell/mm^3^]).^1,4^ Although tecovirimat has shown favourable outcomes in animal studies and case series,^11,12^ high-quality randomised trial data in humans remain limited, and recent trials have not demonstrated clear benefit in immunocompetent adults. In South Africa, access is restricted and requires Section 21 authorisation. Early initiation and extended treatment may be required in immunocompromised patients.^1,4^ Our patient received 14 days of tecovirimat therapy under Section 21 authorisation and displayed good tolerance and clinical improvement, highlighting its potential role as a therapeutic option for mpox in severe disease or immunocompromised states, while acknowledging the limited randomised human efficacy data currently available.

## Conclusion

Mpox is no longer confined to typical high-risk groups. Clinicians must have a broad differential diagnosis, especially in immunocompromised patients with an extensive rash. Early recognition, strict infection control and appropriate therapy, including antivirals like tecovirimat when indicated, are critical to improving outcomes and preventing further transmission.
